# Biomechanical and functional analysis of the shoulder complex and thoracic spine in patients with subacromial impingement syndrome: A case control study

**DOI:** 10.1097/MD.0000000000032760

**Published:** 2023-01-27

**Authors:** Moonyoung Choi, Jinwook Chung

**Affiliations:** a Department of Sports Science Convergence, Dongguk University, Seoul, Republic of Korea.

**Keywords:** muscle strength, scapula, shoulder, subacromial impingement syndrome, thoracic spine

## Abstract

The motions of the shoulder are mainly carried out through the glenohumeral joint, but are also assisted by the scapulothoracic joint. Therefore, changes in the biomechanics of the thoracic spine and scapula affect the function of the shoulder. However, there is limited information on the biomechanical and functional characteristics of the shoulder complex and thoracic spine in patients with subacromial impingement syndrome (SIS). In this study, the biomechanical and functional characteristics of the shoulder complex and thoracic spine were analyzed in patients with SIS compared to healthy individuals. A total of 108 participants were included in this study. Participants were classified into 2 groups, the SIS (n = 55) and healthy (n = 53) groups. The shoulder and thoracic range of motion (ROM), scapular position, and isokinetic shoulder strength were measured in all participants. The shoulder ROM was significantly decreased in the SIS group compared to the healthy group (*P* < .001). The thoracic spine ROM showed significantly limited extension in the SIS group (*P* < .001). The scapular position showed significantly increased anterior tilting (*P* = .005), internal rotation (*P* = .032), protraction (*P* < .001), and decreased upward rotation (*P* = .002) in the SIS group. The isokinetic shoulder external rotation (*P* < .001) and abduction (*P* < .001) strength were significantly lower in the SIS group. Patients with SIS showed reduced shoulder ROM and end-range extension of the thoracic spine compared to healthy individuals, and the scapula was in a more anterior-tilted, protracted, and downward rotated position. In addition, it showed lower external rotation and abduction strength. These results suggest the need for interventions to improve the limited thoracic extension and altered scapular position, which may affect shoulder ROM and muscle strength in the rehabilitation of patients with SIS.

## 1. Introduction

Subacromial impingement syndrome (SIS) is considered the leading cause of shoulder pain, affecting 44% to 65% of all patients with shoulder pain.^[1]^ SIS is a pathological condition in which the subacromial space between the acromion and the humeral head is narrowed, and repeated pressure and the pinching of soft tissues causes pain and dysfunction.^[2]^ The impingement mainly occurs on the anteroinferior surface of the acromion during upper extremity elevation and includes a spectrum of pathologies, such as partial-thickness rotator cuff tear, tendinopathy, calcific tendinitis, and subacromial bursitis.^[3]^

Shoulder motion is mainly carried out through the glenohumeral joint; however, the scapulothoracic joint also contributes to shoulder motion.^[4]^ Unlike the glenohumeral joint that directly connects the upper extremity and trunk by articulating with the humeral head and glenoid fossa of the scapula, the scapulothoracic joint is formed indirectly by the scapula and the thoracic cage.^[5]^ Therefore, changes in the biomechanics of the thoracic spine and scapula affect the function of the shoulder.^[6]^ The scapula’s position and motion are often used as shoulder pathology indicators, and the kinetics of the scapula at the scapulothoracic joint are related to thoracic spine mobility.^[7]^ Limited thoracic mobility alters the relationship between the scapula and humerus, weakening the strength of the shoulder complex. In addition, it causes a limited range of motion (ROM) of the glenohumeral joint, which is a pathological cause of SIS.^[8]^ Bullock et al^[9]^ reported that the active forward flexion angle of the shoulder was increased in the erect sitting posture compared to the slouched sitting posture in a study conducted on patients with SIS. Cools et al^[10]^ analyzed the scapular muscle performance of overhead athletes and found that abnormal muscle action around the shoulder complex caused a change in the position of the scapula and was one of the causes of SIS. Therefore, if a coordinated functional movement with the humerus is not achieved due to abnormal scapular kinetics according to the altered position of the scapula, the risk of shoulder injury increases.^[11]^

Various considerations, such as radiological examination, physical examination, and patient history, have been mainly discussed as methods for diagnosing patients with SIS.^[12,13]^ However, there is limited information on the biomechanical and functional characteristics of the shoulder complex and thoracic spine in patients with SIS. In previous studies, the shoulder ROM and muscle strength characteristics of patients with SIS have been frequently reported. However, there are few studies that compare the thoracic spine ROM and scapular position, which are closely related to shoulder motion and function, in SIS patients and healthy individuals. Therefore, this study aimed to analyze the biomechanical and functional characteristics of the shoulder complex and thoracic spine by comparing shoulder and thoracic spine ROM, scapular position, and isokinetic shoulder strength between patients with SIS and healthy individuals.

## 2. Methods

### 2.1. Participants

This study complied with the Declaration of Helsinki and recruited participants after receiving approval from the institutional review board (approval number: PAIK 2016-08-005). The participants in the SIS group consisted of patients diagnosed with SIS through physical and radiological examinations^[3]^ by an orthopedic surgeon at the same university hospital. The participants in the healthy group were recruited through the hospital bulletin board and website. Both groups included males and females aged between 40 and 60. The number of initially recruited patients with SIS was 102. However, patients with symptoms in both shoulders (n = 20), patients complaining of shoulder pain due to acute traumatic injury (n = 11), patients requiring shoulder surgery (n = 6), patients with limited thoracic mobility due to spinal disease (n = 7), and patients with a history of spine surgery (n = 3) were excluded from the study. Therefore, 55 patients were included in the SIS group in the final analysis. The healthy group included 53 healthy adult males and females who had no symptoms in either shoulder and did not meet the exclusion criteria applied to the SIS group. All participants were fully informed about the purpose and procedure of the study, and voluntarily signed a written informed consent form. In addition, to minimize the effect of variation in activities on the measurements, all participants were restricted from participating in sports or vigorous activities for 24 hours before measurement. The participants underwent goniometer measurement of shoulder ROM, thoracic spine X-ray and scapular computer tomography (CT) examinations, and isokinetic shoulder strength test.

### 2.2. Shoulder range of motion

The shoulder ROM was measured using a goniometer in the supine position.^[14]^ For forward flexion measurement, the lateral center of the humerus head was set as the reference point, and the stationary arm of the goniometer was aligned with the midaxillary line, while the movement arm was aligned with the lateral midline of the humerus. For the abduction measurement, the anterior center of the humerus head was set as the reference point, and the stationary arm of the goniometer was aligned parallel to the sternum, while the movement arm was aligned with the anterior midline of the humerus. The external and internal rotations were measured at 90 degrees of shoulder abduction and 90 degrees of elbow flexion. The goniometer was set with the olecranon process as a reference point so that the stationary arm was aligned vertically to face the ceiling while the movement arm was aligned to face the ulnar styloid. For each measurement, the angle of the endpoint of the maximum active ROM was recorded, and the higher value was recorded by measuring the ROM angles twice.

### 2.3. Thoracic spine range of motion

**Figure 1.**
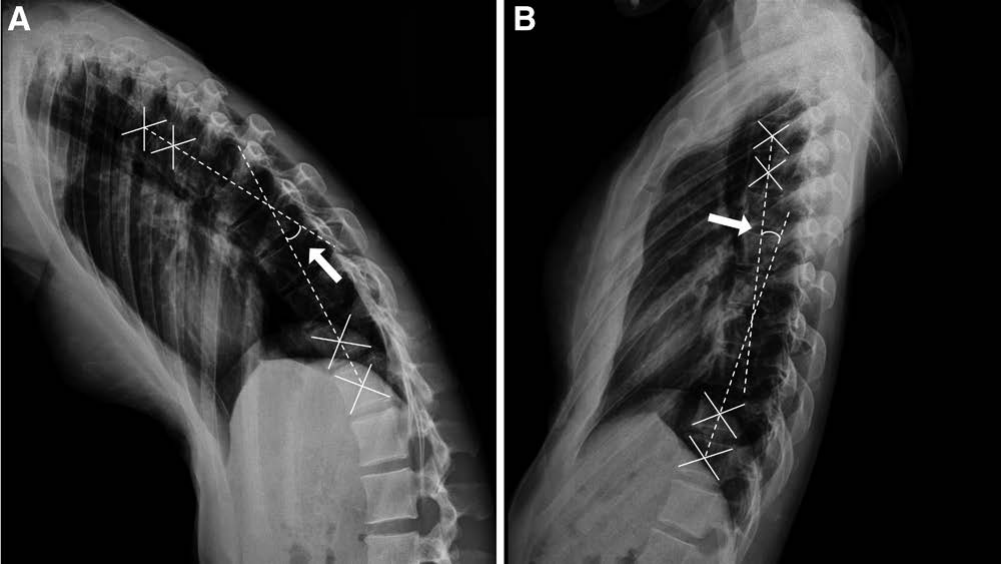
Measurement of thoracic spine range of motion (arrows point to the vertebral centroid angle). (A) Thoracic spine flexion and (B) extension.

The thoracic spine ROM was evaluated through X-ray examination. Lateral thoracic spine radiographs were obtained when the participants’ trunks were in maximal extension and flexion positions. The captured X-ray images were analyzed using m-view 5.4 software (Marotech, Seoul, Korea), a picture archiving and communication system. The vertebral centroid angle was measured by crossing the line crossing the vertebral centroids of thoracic vertebrae 3 to 4 and the line crossing the vertebral centroids of thoracic vertebrae 10 to 11 (Fig. 1).^[15]^

### 2.4. Scapular position

The scapular position was measured using the scapular orientation evaluation method using 3-dimensional wing (3-D wing) CT, which was shown to have high inter-rater reliability in a previous study by Park et al^[16]^ CT scans were performed using a 64-slice multidetector CT scanner (Aquilion TSX-101A, Toshiba Medical Systems Corporation, Otawara, Japan) set at a low radiation dose. The angles of anterior tilting, internal rotation, protraction, superior translation, and upward rotation of the scapula were measured with 3-D wing computer tomography images taken in the resting state using picture archiving and communication system.

#### 2.4.1. Angle definitions.

**Figure 2.**
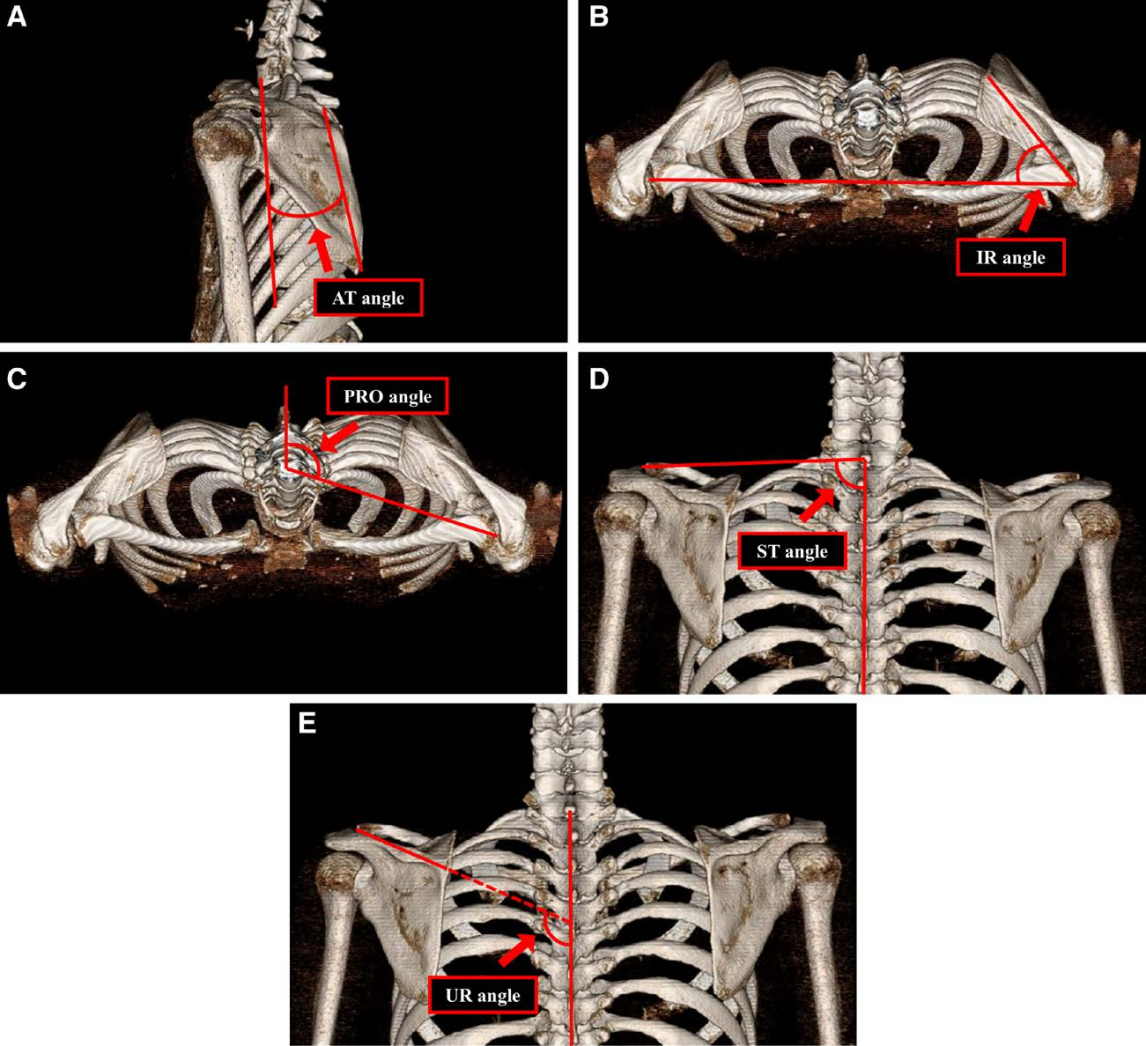
Measurement of scapular position. (A) Anterior tilting (AT), (B) internal rotation (IR), (C) protraction (PRO), (D) superior translation (ST), and (E) upward rotation (UR) angles.

The anterior tilting angle was defined as the angle between the line from the inferomedial angle of the scapula to the medial border and the line connecting the anterior tip of the 7th cervical to the 7th thoracic vertebrae (Fig. 2A).

The internal rotation angle was defined as the angle between the line joining the left and right acromioclavicular joints and the line running from the acromioclavicular joint to the root of the scapular spine (Fig. 2B).

The protraction angle was defined as the angle between the line parallel to the vertebral axis and the line from the center of the vertebral body of the 7th cervical vertebra to the acromioclavicular joint (Fig. 2C).

The superior translation angle was defined as the angle between the line connecting the spinous process of the 7th cervical vertebra at the acromioclavicular joint and the vertebral axis connecting the 7th cervical vertebra to the 7th thoracic vertebra (Fig. 2D).

The upward rotation angle was defined as the angle between the line from the acromioclavicular joint through the root of the scapular spine to the spine and the line from the 7th cervical vertebra to the spinous process of the 7th thoracic vertebra (Fig. 2E).

### 2.5. Isokinetic shoulder strength

The isokinetic shoulder strength test was performed using an isokinetic dynamometer (HUMAC NORM, CSMi, MA), and external rotation, internal rotation, abduction, and adduction strength of both shoulders were measured.^[17]^ The participants sat in the examination chair and matched the axis of the dynamometer with the anatomical axis of the shoulder to maintain a consistent examination posture. In addition, the pelvis and trunk were fixed with straps to minimize compensatory movements. The test method was sufficiently explained, and several pre-practice sessions were conducted to help the participants understand the examination. The ROM for the rotations of the shoulder was set to 50 degrees external rotation and 30 degrees internal rotation from the neutral position after fixing the elbow to the dynamometer (total ROM was 80 degrees). The ROM for abduction and adduction was set from the anatomical position (0 degrees) to 70 degrees of abduction after the dynamometer was aligned parallel to the scapular plane. The participants repeated each test 4 times at an angular velocity of 60 degrees/second. For the analysis, the peak torque values of both shoulder muscles were measured. The Limb symmetry index was calculated using the measured peak torque values to compare the muscle strength ratio between both shoulders. For comparison with the SIS group, the healthy group defined the non-dominant shoulder as the involved limb and the dominant shoulder as the uninvolved limb. The Limb symmetry index of both shoulder strengths was calculated as follows:

Limb symmetry index = (Involved limb/Uninvolved limb) × 100.

### 2.6. Statistical analysis

The sample size was calculated using G*Power 3.1 (University of Düsseldorf, Düsseldorf, Germany). The conditions were as follows: effect size = 0.5, alpha level = 0.05, power = 0.80. The statistical analysis was performed using SPSS 22.0 (IBM Corp., Armonk, NY). Continuous variables are expressed as means and standard deviations, and categorical variables are expressed as numbers and percentages. Normality tests were performed using the Kolmogorov–Smirnov and Shapiro–Wilk tests. The main variables were not normally distributed. Therefore, a non-parametric analysis was performed. The Mann–Whitney *U* test was used to compare the differences between the 2 groups. Statistical significance was set at *P* < .05.

## 3. Results

### 3.1. General characteristics of participants

The participants were classified according to the groups and analyzed. The general characteristics are shown in Table 1. Age, height, weight, Body mass index, and sex were not significantly different between the groups.

**Table 1:** General characteristics of participants.

Variables	SIS (n = 55)	Healthy (n = 53)	*t* or *χ*^2^	*P* value
Age, yr	50.05 ± 4.24	50.32 ± 3.42	−0.358	.721
Height, cm	168.80 ± 5.59	169.74 ± 5.98	−0.844	.401
Weight, kg	68.14 ± 7.31	67.67 ± 8.31	0.311	.756
BMI, kg/m^2^	23.84 ± 1.60	23.39 ± 1.67	1.435	.154
Sex, n (%)				
Male	29 (52.7)	30 (56.6)	0.164	.686
Female	26 (47.3)	23 (43.4)		

Values are expressed as the mean ± standard deviation or number (%).

BMI = body mass index, SIS = subacromial impingement syndrome.

### 3.2. Differences in shoulder range of motion between groups

Table 2 shows the differences between groups in shoulder ROM. Compared with the healthy group, the SIS group showed significantly reduced ROM in forward flexion (*P* < .001), abduction (*P* < .001), and external (*P* < .001) and internal rotations (*P* < .001) of the shoulder.

**Table 2:** Comparative analysis of shoulder range of motion between groups.

Variables	SIS	Healthy	Difference (%)	*P* value
Forward flexion, degree	165.82 ± 4.56	175.17 ± 2.95	5.6	<.001*
Abduction, degree	162.36 ± 8.16	172.72 ± 2.86	6.4	<.001*
External rotation, degree	60.04 ± 4.96	68.58 ± 4.17	14.2	<.001*
Internal rotation, degree	51.36 ± 5.34	58.64 ± 4.06	14.2	<.001*

Values are expressed as the mean ± standard deviation.

SIS = subacromial impingement syndrome.

* Statistically significant (*P* < .05).

### 3.3. Differences in thoracic spine range of motion between groups

Table 3 shows the differences between groups in thoracic spine ROM. The SIS group showed significantly reduced ROM in thoracic spine extension (*P* < .001) and total ROM (*P* < .001) compared with the healthy group. However, there was no significant difference in flexion between the groups.

**Table 3:** Comparative analysis of thoracic spine range of motion between groups.

Variables	SIS	Healthy	Difference (%)	*P* value
Flexion, degree	32.25 ± 1.93	31.70 ± 2.37	1.7	.157
Extension, degree	16.97 ± 1.34	13.17 ± 1.31	22.4	<.001*
Total ROM, degree	16.28 ± 2.33	18.53 ± 2.68	13.8	<.001*

Values are expressed as the mean ± standard deviation.

ROM = range of motion, SIS = subacromial impingement syndrome.

* Statistically significant (*P* < .05).

### 3.4. Differences in scapular position between groups

Table 4 shows the differences between groups in the scapular position. The SIS group showed significantly increased anterior tilting (*P* = .005), internal rotation (*P* = .032), and protraction (*P* < .001) angles, and decreased upward rotation (*P* = .002) angle compared with the healthy group. However, the superior translation angle did not differ significantly between the groups.

**Table 4:** Comparative analysis of scapular position between groups.

Variables	SIS	Healthy	Difference (%)	*P* value
AT angle, degree	7.21 ± 2.45	5.90 ± 1.39	18.2	.005*
IR angle, degree	51.17 ± 4.00	49.48 ± 3.49	3.3	.032*
PRO angle, degree	103.53 ± 3.16	100.87 ± 2.95	2.6	<.001*
ST angle, degree	91.85 ± 2.59	91.21 ± 1.71	0.7	.139
UR angle, degree	111.51 ± 5.11	114.21 ± 3.46	2.4	.002*

Values are expressed as the mean ± standard deviation.

AT = anterior tilting, IR = internal rotation, PRO = protraction, SIS = subacromial impingement syndrome, ST = superior translation, UR = upward rotation.

* Statistically significant (*P* < .05).

### 3.5. Differences in isokinetic shoulder strength between groups

Table 5 shows the differences between groups in isokinetic shoulder strength. Compared with the healthy group, the SIS group showed significantly lower muscle strength in external rotation (*P* < .001) and abduction (*P* < .001). However, there was no significant difference in strength between groups in internal rotation and adduction.

**Table 5:** Comparative analysis of isokinetic shoulder strength between groups.

Variables	SIS	Healthy	Difference (%)	*P*-value
External rotation, %†	82.04 ± 2.80	95.24 ± 2.21	16.1	<.001*
Internal rotation, %†	92.86 ± 5.82	94.34 ± 2.58	1.6	.363
Abduction, %†	80.73 ± 3.66	95.93 ± 2.08	18.8	<.001*
Adduction, %†	95.19 ± 3.33	96.21 ± 2.44	1.1	.412

Values are expressed as the mean ± standard deviation.

SIS = shoulder impingement syndrome.

* Statistically significant (*P* < .05).

† Limb symmetry index.

## 4. Discussion

Shoulder pain, limited ROM, and muscle weakness are typical symptoms that frequently occur in patients with SIS and lead to sleep disturbance and reduced physical activity, lowering the quality of life.^[18]^ The limited mobility of the thoracic spine, among other factors, can lead to or exacerbate SIS by inappropriately altering the orientation and motion of the scapula.^[8]^ The biomechanics and function of the shoulder are closely related to the thoracic spine and scapula, but the characteristics of thoracic mobility and scapular position in patients with SIS have not been investigated in detail. Therefore, in this study, the biomechanical and functional characteristics of SIS patients and the healthy controls were compared and analyzed by evaluating shoulder and thoracic spine ROM, scapular position, and isokinetic shoulder strength.

In this study, patients with SIS showed more reduced forward shoulder flexion, abduction, external rotation, and more limited thoracic spine extension compared to healthy individuals. Previous studies have also tried to identify the causes of the limited shoulder ROM in patients with SIS.^[19,20]^ The human shoulder involves complex mechanisms that enable a wide range of movements and provide the mobility and structural support needed to perform daily activities and sports.^[21]^ The role of the thoracic spine in influencing these shoulder biomechanics has been investigated by several authors.^[22,23]^ During spinal alignment, the thoracic spine is significantly involved in shoulder motion. During unilateral arm elevation in the sagittal and the scapular planes, coupled movements of lateral flexion and rotation of the upper thoracic spine appear ipsilaterally, and these movements are related to the extension of the thoracic spine.^[24]^ In addition, thoracic kyphosis due to increased thoracic flexion in the resting position may alter the relationship between the scapula and the humerus. These alterations may lead to limitations in shoulder ROM and, consequently, the pathology of SIS.^[25]^

In this study, the scapular position in the resting posture showed greater anterior tilting, internal rotation, protraction, and downward rotation in patients with SIS compared to healthy individuals. The characteristics of the scapular position in patients with SIS observed in this study are consistent with those reported in previous studies. Turgut et al observed asymmetry of scapular kinematics between the involved and uninvolved shoulder in patients with SIS and reported increased protraction, downward rotation, and anterior tilting of the scapula in the involved shoulder.^[26]^ In this study, patients with SIS showed combined asymmetry of the scapular position in the resting posture and limited extension of the thoracic spine during dynamic motion. In the biomechanics of the shoulder complex, upward rotation of the scapula for the functional orientation of the glenoid fossa is important to increase the dynamic stability of the glenohumeral joint during upper extremity elevation.^[27]^ Thigpen et al reported increased upward rotation and decreased internal rotation of the scapula in the straightened thoracic spine posture than in the forward head and rounded shoulder postures.^[28]^ Limited thoracic extension can compensate to increase the kyphosis of the upper thoracic spine in the resting position.^[29]^ Patients with SIS exhibited more limited thoracic spine extension than healthy individuals. This characteristic may be one of the causes of the scapular position change toward an inappropriate orientation by increasing the thoracic kyphosis in the resting posture. On the other hand, scapular elevation is more sensitively affected by dynamic factors such as tension of the scapular elevation muscles including upper trapezius and levator scapulae than static factors such as limited mobility of the thoracic spine.^[30]^ Since all participants in this study relaxed their muscles as much as possible in the resting posture and measured the position of the scapula, it is thought that the superior translation angle did not show a significant difference between the groups in the analyzed results.

Previous studies have tried to objectively evaluate the relationship between shoulder kinetics and shoulder muscle strength in patients with SIS.^[31,32]^ Most of the authors found kinetic changes in the scapula during dynamic shoulder motion and reported that these changes were related to muscle weakness and imbalance of the rotator cuff muscles.^[31,33]^ In this study, patients with SIS showed lower external rotation and abduction strength than healthy individuals. However, there was no significant difference in internal rotation and adduction muscle strength. The internal rotator and adductor muscles of the shoulder are not directly affected by pathological impingement that occurs in the narrowed subacromial space due to the characteristics of their anatomical location.^[34]^ Therefore, the isokinetic strength test performed in a controlled motion and range detected a sensitively significant difference only in the external rotator and abductor muscles of the shoulder. This characteristic of muscle strength in patients with SIS may be related to increased thoracic kyphosis due to limited thoracic spine extension. Thoracic kyphosis is directly related to the narrow subacromial space and may indirectly affect the development of SIS by reducing upper extremity elevation due to thoracic spine extension restriction and scapular dyskinesis.^[25]^ In addition, a rounded shoulder posture caused by thoracic kyphosis has been reported as the cause of inappropriately changing the scapular position.^[35]^ In this state, repeated and excessive use of the shoulder causes pain and inflammation in the subacromial soft tissues, which leads to weakness and imbalance of the shoulder muscle.^[36]^ It is thought that changes in the biomechanics of the shoulder complex related to the scapulothoracic joint causes pressure and impact on the rotator cuff tendons involved in shoulder abduction and external rotation, leading to muscle weakness.

This study has some limitations, including the relatively small sample size and the old age of the participants. Since only the characteristics of middle-aged adults between 40 and 60 years of age were investigated in this study, it is necessary to investigate the biomechanical and functional characteristics of young patients with SIS in future studies. Also, due to the inherent limitations of the 3-D wing computer tomography evaluation method that evaluates the position of the scapula in a static position, the dynamic position of the scapula may not be properly reflected. In order to overcome this limitation, efforts were made to measure the position of the scapula as accurately as possible in the resting position in which the periscapular muscles were relaxed.

## 5. Conclusion

This study showed that patients with SIS exhibited reduced shoulder ROM and end-range extension of the thoracic spine compared to healthy individuals and a scapula that was in a more anterior-tilted, protracted, and downward rotated position. In addition, this study showed lower external rotation and abduction strength in patients with SIS compared to healthy controls. These results suggest the need for interventions to improve limited thoracic extension and altered scapular position, which may affect shoulder ROM and muscle strength in the rehabilitation of patients with SIS.

## Acknowledgments

We would like to thank all of the participants for their time and commitment to the present study.

## Author contributions

**Conceptualization:** Moonyoung Choi, Jinwook Chung.

**Data curation:** Moonyoung Choi.

**Formal analysis:** Moonyoung Choi, Jinwook Chung.

**Investigation:** Moonyoung Choi.

**Methodology:** Moonyoung Choi, Jinwook Chung.

**Supervision:** Jinwook Chung.

**Writing – original data:** Moonyoung Choi.

**Writing – review & editing:** Jinwook Chung.
